# Does the Order of Submaximal Lactate Threshold and Maximal Oxygen Uptake Testing Influence Test Outcomes?

**DOI:** 10.3390/sports8060075

**Published:** 2020-05-26

**Authors:** Per-Øyvind Torvik, Roland van den Tillaar, Gaute Iversen

**Affiliations:** Department of Sports Sciences and Physical Education, Nord University, 7600 Levanger, Norway; roland.v.tillaar@nord.no (R.v.d.T.); gaupaiversen@hotmail.com (G.I.)

**Keywords:** test protocol, maximal oxygen uptake, lactate threshold, exercise physiology

## Abstract

The aim of this study was to investigate if the order of submaximal lactate threshold and maximal oxygen uptake testing would influence test outcomes. Twelve well-trained male cross-country skiers (mean age 19.6 years) performed two test sessions within a week in a within-subjects repeated measures with cross-over design study. A maximal oxygen uptake test (VO_2max_) followed by a lactate threshold (LT) test and vice versa, were performed. The test data included VO_2_, blood lactate (La^-^_b_), heart rate (HR), performance speed, Borg scale (RPE) at all stages and lactate accumulation throughout the whole test protocol including the breaks. No significant effect of testing order was found for: VO_2max_ (74.23 vs. 73.91 mL∙min^−1^∙kg^−1^), maximal HR (190.7 vs. 189.9 bpm) and speed at LT during uphill running. Three out of four common definitions of LT resulted in the same La^-^_b_ at the last two steps, 11 and 12 km/h respectively, in the two protocols. It is worth noting that VO_2_, HR and La^-^_b_ were higher in the first two stages of the LT test when VO_2max_ was tested first in the protocol. Well-trained cross-country skiers conclusively attained a similar VO_2max_ and LT in both protocols, and the two tests did not seem to influence each other in terms of the degree of exhaustion that occurs in a single VO_2max_ or an incremental LT test. However, when using a curvilinear function to define the LT, it is important to know that the VO_2max_ test can influence levels of VO_2_, HR and La^-^_b_ at the first two stages of the LT test.

## 1. Introduction

Maximal oxygen uptake (VO_2max_) and lactate threshold (LT) are often used to evaluate the physiological effect of endurance training and to predict performance in endurance sports for elite athletes [1,2]. These are generally tested in the laboratory to ensure that the measured parameters are both valid and reliable. Research on VO_2max_ and LT has been ongoing for almost a century and has been reviewed regularly; as a result, VO_2max_ and LT have been used as predictors of endurance performance [3,4,5]. VO_2max_ is not only a measure of aerobic energy turnover, but is also considered to be a precise measure of lung capacity, heart rate and oxygen transport capacity of the blood, and the ability of muscles and mitochondria to utilize oxygen [6].

LT is normally tested on a treadmill or in a specific movement pattern and is often used to monitor the training process and specific performance [7]. The concept demonstrates that lactate accumulation occurs later (shifting to a higher percentage of VO_2max_, at a lower heart rate and a higher workload) after a period of endurance training. In athletes, the level of work intensity that can be sustained for an extended period prior to lactate accumulation is an accurate predictor of endurance performance [8]. In order to obtain steady state aerobic conditions, the stages used to assess LT should be longer than 4 min, and beyond the duration of stages used for incremental testing of VO_2max_. Thus, LT and VO_2max_ are normally determined in two separate tests.

Testing elite athletes must be time effective and well-organized to prevent undue interference with their training schedule. Therefore, the two separate tests for determining LT and VO_2max_ are often done on the same day. It is often difficult to organize scientific testing and training evaluation over several days, due to the influence of the elite athletes’ own training schedule.

The nature of one test, due to the degree of exhaustion and motivation, is hypothesized to affect results in the next test in the protocol with insufficient recovery. When testing VO_2max_, the procedure in much scientific work and many test labs requires no exhausting activity the day before or the same day since this is expected to negatively influence the result [9]. This also applies to the testing of LT, and it is hypothesized that an exhausting VO_2max_ test immediately before the test will negatively influence the La^-^_b_ values and LT speed. However, to our knowledge there is no scientific evidence to support the hypothesis that endurance tests will influence test outcome in the following test. The study aim was therefore to investigate the effect of the order of submaximal LT and VO_2max_ testing on test outcomes.

## 2. Materials and Methods

### 2.1. Methods

A within-subject repeated measures design with cross-over was used to investigate the effect of the order of submaximal LT and VO_2max_ testing on test outcomes.

### 2.2. Subjects

The subjects in this study were twelve well-trained male cross-country skiers (mean age, SD 20.0 ± 1.7 years, body mass 71.5 ± 5.3 kg, height 1.81 ± 0.06 m). The participants were athletes that had trained and competed in cross-country skiing for 6–10 years and had competed at a national level for at least four years. All participants were familiar with running on a treadmill and the test protocols for VO_2max_ and LT. Inclusion criteria were having a VO_2max_ above 65 mL∙min^−1^∙kg^−1^. All participants were 18 years or older at the start of the study. All participants received written information about the entire content of the study and signed an informed consent prior to voluntary participation in the study. They were informed of their right to withdraw from the study at any time. The study was approved by the Norwegian Center for Research Data (NSD).

### 2.3. Procedures

To study whether the two tests influenced each other, the subjects performed the two test protocols within 48 h to 7 days. The order of each test combination was a within-subjects repeated measure with cross-over design, i.e., a VO_2max_ test before an LT (VO_2max_ -LT) or an LT test before the VO_2max_ test (LT-VO_2max_). The athletes were tested in the lab on two occasions, at least 48 h apart. They were instructed to prepare as if they were going to participate in a competition, during the last 24 h before the test. All the athletes were to arrive well rested and hydrated, and all were given a standard light meal consisting of oat porridge topped with sugar, cinnamon and butter and apple juice, estimated at 285 Kcal, two hours before testing.

The skiers had 20 min with active recovery between the two tests. La^-^_b_ was monitored during the active recovery phase between the two tests in both protocols at 15, 10, 5 and 0 min before the LT test or VO_2max_ test. The test protocols contained a 15 min warm-up at 60% of VO_2max_, followed by a measurement of La^-^_b_ in the fingertip (La^-^_b_); this La^-^_b_ value was taken as a baseline from which the LT test was calculated [10].

The VO_2max_ test was an incremental uphill running test with increasing speed by 1 km h^−1^ every min until exhaustion at 10.5% inclination on a 2.5 × 0.7 m motor-driven treadmill (RL 2500E, Rodby, Södertalje, Sweden). Gas exchange was measured continuously and VO_2max_ was defined if two out of the following three criteria were reached: (1) attaining a plateau in VO_2_ despite increased intensity, (2) respiratory exchange ratio (RER) > 1.10, and (3) peak La^-^_b_ > 8 mmol/L [11]. The LT test was also an incremental uphill treadmill running test, but with increasing speed by 1 km h^−1^ every five min with a standard break of one min for recording of RPE, end heart rate, La^-^_b_ and increase in the speed on the treadmill. LT was defined as the running speed when the subject’s La^-^_b_ increased by ≈ 4.0 mmol/L or by more than 1.0 mmol/L between two intervals [12]. A visual control of the break point where La^-^_b_ increased exponentially was also conducted. Several laboratories use a detection method that includes warm-up values of +1.5 mmol/L [13], while others use a fixed 4.0 mmol/L [14]. Researchers have tried to establish an accurate measure of LT [15] and there is an accepted concept of a right shift of the curve as a sign of improved LT level, but there are no generally accepted values or methods for detecting the LT itself. To avoid fatigue, the LT-VO_2max_ test was aborted when the subjects had reached an La^-^_b_ of 4 mmol/L, which has been used as the absolute highest value of LT testing [12]. To investigate the physical response to the test protocol, VO_2_ was measured during the last 3 min of every interval in the LT test. The subjects wore a nose clip and a mouthpiece for the VO_2_ measurement during the whole interval, while running.

### 2.4. Measurements

La^-^_b_ were determined from 20-μL samples from the fingertip and analyzed by the BIOSEN C-line Sport (EKF Diagnostic, Magdeburg. Germany). HR was measured with a heart rate monitor (Polar RC3GPS, Polar Electro OY, Kempele, Finland), using a five-second interval for data storage. Body mass was measured with an electronic body mass scale (Seca model no. 708, Seca GmbH & Co, Hamburg, Germany) and height with a stadiometer (Holtain Ltd., Crosswell, UK). Rating of perceived exertion (RPE) was recorded using the 6–20 point Borg Scale [16].

Oxygen uptake was measured by an Oxycon Pro with a mixing chamber (Jaeger GmbH, Höchberg, Germany), and the mean of the three highest consecutive 10-sec measurements of VO_2_ at each stage was designated as VO_2peak_ [1]. At the start of each fifth test daily, the VO_2_ and VCO_2_ gas analyzers were calibrated against both ambient air and a commercial mixture of high-precision gases (15.920 ± 0.04% O_2_ and 5.030 ± 0.1% CO_2_, CareFusion Gas GmbH, Höchberg, Germany). The O_2_ and CO_2_ content of the ambient air was recorded, and the flow meter was calibrated with a 3-L high-precision syringe (Hans Rudolph Inc., Kansas City, Missouri, USA). A Polar RC3GPS (Polar Electro OY, Kempele. Finland) was used to measured HR every five sec. Calibration of the La^-^_b_ analyzer was performed every hour and checked using a standard of 12 mmol/L. Voltage was assessed using a Redicom Norm solution of 2.69–3.35 mmol/L.

### 2.5. Statistical Analysis

All data were controlled for normality and the Shapiro–Wilk test concluded that the data were normally distributed. No data were excluded from the study. One and two-way ANOVA with repeated measurements on test order were used for the physiological and perceptual responses during and at the end of the LT and VO_2max_ tests. Post-hoc comparisons with Holm–Bonferroni corrections were conducted to determine differences. When sphericity assumptions were violated, Greenhouse–Geisser adjustments of the *p*-values were reported. The effect size used and reported in this study was partial eta squared (η^2^), where 0.01 ≤ η^2^ < 0.06 constituted a small effect, 0.06 ≤ η^2^ < 0.14 constituted a medium effect, and η^2^ < 0.14 constituted a large effect (Cohen, 1988). The level of significance was set at *p* ≤ 0.05 for all tests and the analyses were carried out with SPSS Statistics v24 (SPSS Inc., Chicago, IL, USA). To assess reliability of the performances (time to exhaustion) and dependent physiological responses (VO_2max_, heart rate) at the end of both tests in each order, the ICC (2,1) and the coefficient of variation (CV) were calculated.

## 3. Results

The HR (132 ± 11 vs. 131 ± 12 beats/min) and La^-^_b_ values (1.15 ± 0.30 vs. 1.25 ± 0.39 mmol/L) immediately after the warming up were not significantly different before starting the two test occasions.

**Figure 1:**
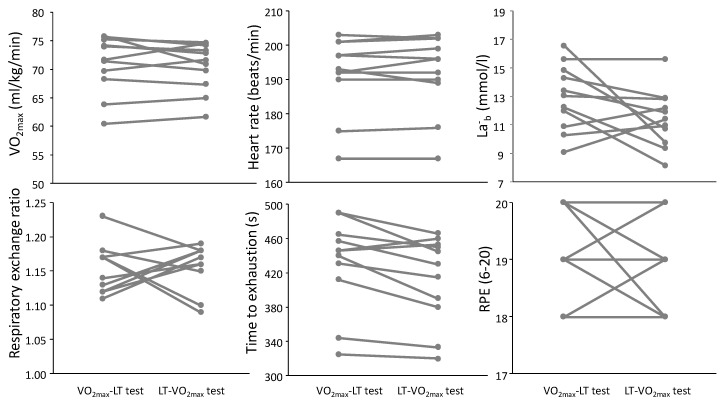
Individual physiological responses at the end of the uphill running VO_2max_ test at a fixed inclination of 10% at the two different conditions: VO_2max_ test before LT test or the opposite: LT before VO_2max_.

**Figure 2:**
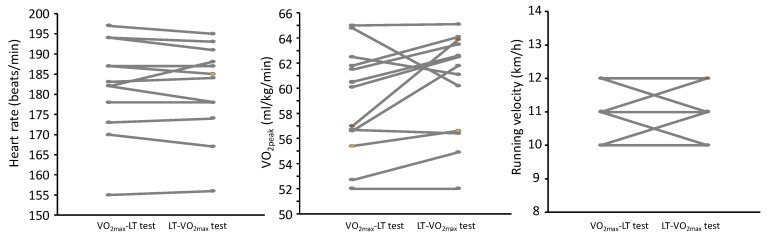
Individual physiological responses and running velocity at the end of the lactate threshold test (La^-^_b_ values ≈ 4 mmol/L) at the two different conditions: VO_2max_ test before LT test or the opposite: LT before VO_2max._

No significant differences were found due to the testing order of the VO_2max_ test (Table 1 and Figure 1). In the VO_2max_ test, no significant differences between test conditions were found for any of the physiological and perceptual responses (F ≤ 3.04, *p* ≥ 0.107, η^2^ ≤ 0.20). At the end of the LT test (Table 2 and Figure 2), there were also no significant differences for any of the physiological and perceptual responses (F ≤ 3.04, *p* ≥ 0.105, η^2^ ≤ 0.20). Furthermore, the ICCs varied from 0.87 (VO_2peak_ at end LT test) to 0.99 (HR at end of VO_2max_ and LT tests) with CVs varying from 0.62 to 3.9%.

**Figure 3:**
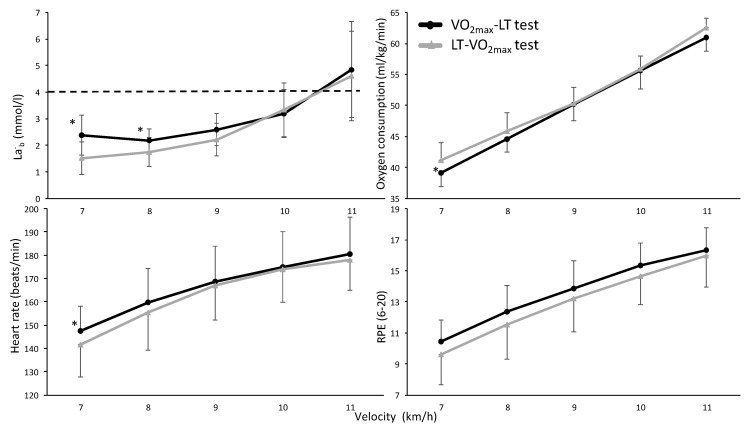
Mean (±SD) La^-^_b_, oxygen consumption, HR and RPE at the different running velocities at the different testing orders (LT-VO_2max_ or VO_2max_-LT) with the fixed 4 mmol/L La^-^_b_ level. * indicates a significant difference between these two variables at this velocity on a *p* < 0.05 level.

A significant effect was found for all physiological and perceptual parameters during the LT test (F ≥ 26.2, *p* ≤ 0.001, η^2^ ≥ 0.79); all parameters increased with increasing running velocity (Figure 3). No significant effect for testing order (F ≤ 1.28, *p* ≥ 0.30, η^2^ ≤ 0.15) or interaction effect (F ≤ 1.9, *p* ≥ 0.125, η^2^ ≤ 0.22) was found for these parameters. However, post hoc comparison revealed lower HR, La^-^_b_ and oxygen uptake at 7 km/h when starting with the LT test than when starting with the VO_2max_ test. In addition, at 8 km/h the La^-^_b_ values were still lower in the LT-VO_2max_ test order compared with the VO_2max_-LT test order (Figure 3).

**Figure 4:**
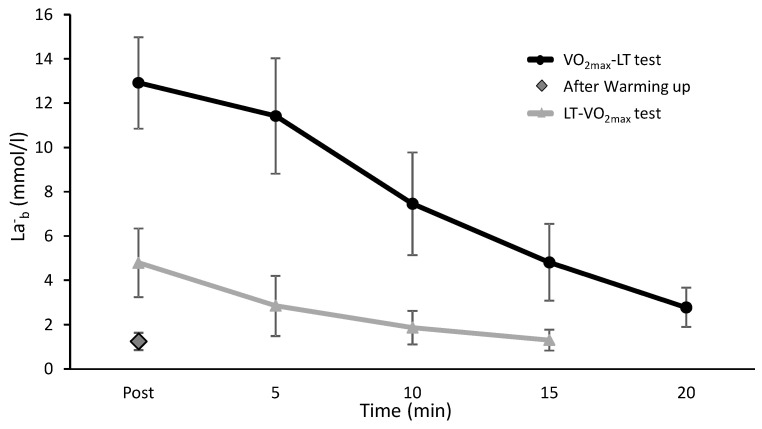
Mean (±SD) La^-^_b_ concentration straight after the warm-up and after the first test of the LT-VO_2max_ and VO_2max_-LT protocols.

La^-^_b_ concentrations after completing the first tests were 12.9 ± 2.1 and 4.8 ± 1.6 after the VO_2max_ and LT tests and decreased significantly every five min. Fifteen min after conducting the LT test, La^-^_b_ had reached a similar level to just after the warm-up, while the La^-^_b_ concentration after the VO_2max_ test still had not reached the values of immediately after the warm-up (Figure 4). However, no left or right shift of the La^-^_b_ curve was observed.

## 4. Discussion

This study investigated the effect of testing order of the VO_2max_ and LT test in the same session upon these aspects of performance in these two tests. Our main findings, for well-trained cross-country skiers, were that no physiological differences were found due to the testing order. The exception was at the start of the LT test after conducting the VO_2max_-LT protocol first, where the physiological parameters tested were higher than in the opposite testing order. Furthermore, the La^-^_b_ concentration 20 min after the VO_2max_ test did not reach similarly low levels to the warm-up, while after 15 min of the LT-VO_2max_ protocol, the La^-^_b_ levels were similar to those just after the warming up session. This resulted in higher values (La^-^_b_, HR and VO_2_) in the two first steps of the VO_2max_-LT protocol, without any effect on the speed at LT.

The results of this study do not support the recommendation given in the testing methodology concerning the effect of having exhaustive activity the same day or the day before. These recommendations take all precautions into consideration, such as the level of the athlete, sex, age and body composition. However, the subjects in this study, well-trained cross-country skiers, were not significantly affected by either of the two protocols on reaching VO_2max_ or establishing the level of LT. Their ability to push themselves to exhaustion may be dependent on the fact that they are used to training for long periods, with several training sessions per day, compared to untrained subjects. However, the ability to reach physical parameters is reported to be independent of physical training status [17]. By contrast, Berglund et al. [18] speculate that fit individuals are better at pushing themselves to exhaustion than unfit individuals. Athletes at a lower physical activation level would probably have greater difficulty with a submaximal test after a maximal test in one test session. However, the RPE were slightly, but not significantly, higher (0.6–0.8) for the first two steps of the VO_2max_-LT protocol compared to the LT-VO_2max_ protocol. Despite 20 min of active recovery after a VO_2max_ test, one may expect to feel a little more exhausted than without. These findings indicate that most healthy, well-trained elite cross-country skiers reach similar values for LT and VO_2max_ independently of the two test protocols despite the slightly higher RPE in the first two steps.

In both test conditions, all subjects achieved the criteria for VO_2max_ and there was no significant difference in VO_2max_ between HR_peak_, La^-^_b_, RER and reaching a plateau in VO_2_ despite increasing workload. The subjects also reported the same subjective exhaustion (RPE). It is also to be noted that the subjects had the same running speed and time to exhaustion. The difference was also within the expected day to day variation of 2 mL∙min^−1^∙kg^−1^. The level of VO_2max_ is considered to be a relatively rough measure of endurance capacity; the measure is relatively stable within the variance of the equipment and day to day variation and quite easy to reach [11]. Our findings show that VO_2max_ is not affected by previous activity in trained athletes and that they reach their VO_2max_ in both test protocols. This test on well-trained athletes is not affected by activity at and just over the LT on the same day.

However, it must be considered that a normal improvement of quality endurance training in VO_2max_ per year, in well-trained athletes, is reported to be 2 mL∙min^−1^∙kg^−1^ [19]. This indicates the importance of accuracy in the test procedure, if the aim is to measure the effect of endurance training on VO_2max_ rather than to establish a level of VO_2max_.

We assume that if the most important goal is to evaluate the effect of training on the level of VO_2max_ and the athlete and the researcher have limited time and resources in the test conditions, an VO_2max_-LT protocol is preferable. To prevent any form of bias, the best protocol in research is probably a plain VO_2max_ test.

The speed at LT seems to be unaffected by a prior exhausting VO_2max_ in well-trained cross-country skiers. The La^-^_b_ values were higher at the two first velocity levels of the VO_2max_-LT protocol. The VO_2_ and HR were also significantly higher at the first level. However, there were no significantly higher RPE values at these two workloads. The break following the VO_2max_ test was 20 min, containing easy walking at 4 km/h, 10% inclination, resting and preparing actively for the LT protocol. During this time the La^-^_b_ values declined from an average of 11.37 mmol/L to 2.78 mmol/L, but not down to warm-up level. La^-^_b_ continued to decrease to 2.25 mmol/L at the end of the first step, and further down to 2.07 mmol/L after step two. This indicates that the active recovery and the workloads at the two first steps in the VO_2max_-LT protocol brought the La^-^_b_ values down to the level below LT values at steps three and four. At step three, the La^-^_b_ values were identical (3.02 mmol/L) in the two protocols. The different methods to determine LT (4 mmol/L, break point or first workload that elevated La^-^_b_ values more than 1 mmol/L between the two steps) did not correspond with different speeds or oxygen uptake between protocols (LT-VO_2max_ vs. VO_2max_-LT). There was also no right or left shift of the La^-^_b_ curve due to protocol, which would have been proof of an effect of the test order. If the use of a warming up La^-^_b_ value of + 1.5 mmol/L was used, this would give different detection of the LT La^-^_b_ values (3.0 and 3.8 mmol/L) due to the higher warming up values in the VO_2max_-LT protocol. In that way, the LT test would be stopped at a lower running speed in the VO_2max_-LT protocol than in the LT-VO_2max_ (10.9 vs. 10.5 km/h in the present study). Thereby, the VO_2max_-LT protocol would underestimate the LT, while it was shown in the present study that in the later stages of the LT test, in both protocols, the same development and values were observed. This indicates that the 4.0 mmol/L fixed method combined with the more than 1.0 mmol/L increase of La^-^_b_ between two speed intervals is perhaps a better method to use when performing a LT and VO_2max_ test after each other. It must also be considered that the LT measurements also tend to show higher day-to-day variation than VO_2max_ and HR_max_ [20]. No such observation was made in our study.

## 5. Conclusions

Most previous protocols for testing of LT and VO_2max_ have adopted a standard of 24 to 48 h recovery time. The practice undertaken in this study of coupling VO_2max_ and LT tests in the same test protocol for well-trained athletes worked well. The order is irrelevant, assuming that enough recovery and an active break between the tests is provided. It is also recommended that the break in the VO_2max_-LT protocol is longer than the break in the LT-VO_2max_ protocol, e.g., 20 vs. 15 min. The LT-VO_2max_ protocol has several advantages, such as shorter total use of time and a less demanding test overall, if the LT test is stopped around 4 mmol/L. However, it would seem important to use the same protocol from time to time to prevent measurement or systematic errors.

## Figures and Tables

**Table 1:** Physiological responses at the end of the uphill running VO_2max_ test at a fixed inclination of 10% at the two different conditions: VO_2max_ test before LT test or the opposite: LT before VO_2max_ and *p* value between the two tests for each variable.

Test Order	VO_2max_ (mL/min/kg)	Heart Rate (Beats/min)	Respiratory Exchange Ratio	Time to Exhaustion (s)	La^-^_b_ (mmol/L)	RPE
VO_2max_-LT	71.5 ± 4.6	192.1 ± 10.1	1.16 ± 0.03	442 ± 37	12.9 ± 2.0	19.2 ± 0.7
LT-VO_2max_	71.3 ± 4.2	192.3 ± 10.0	1.16 ± 0.03	429 ± 40	11.7 ± 1.6	19.1 ± 0.8
*p* value	0.48	0.90	0.95	0.13	0.11	0.34

**Table 2:** Physiological responses at the end of the LT test at the two different conditions and *p* value between the two tests for each variable.

Test Order	VO_2max_ (mL/min/kg)	Heart Rate (beats/min)	Velocity at ≈ 4 mmol/L (km/h)	La^-^_b_ (mmol/L)	RPE
VO_2max_-LT	59.5 ± 3.9	181.0 ± 11	11.0 ± 0.8	4.65 ± 1.52	16.2 ± 1.2
LT-VO_2max_	60.8 ± 3.8	180.2 ± 11	11.2 ± 0.8	4.48 ± 1.5	16.2 ± 1.7
*p* value	0.11	0.60	0.72	0.53	1.00

## References

[1] Bassett D.R. (2000). Limiting factors for maximum oxygen uptake and determinants of endurance performance. Med. Sci. Sports Exerc..

[2] Levine B.D. (2008). VO2max: What do we know, and what do we still need to know?. J. Physiol..

[3] Ingjer F. (2007). Maximal oxygen uptake as a predictor of performance ability in women and men elite cross-country skiers. Scand. J. Med. Sci. Sports.

[4] Mahood N.V., Kenefick R.W., Kertzer R., Quinn T.J. (2001). Physiological determinants of cross-country ski racing performance. Med. Sci. Sports Exerc..

[5] Sandbakk O., Holmberg H.-C., Leirdal S., Ettema G. (2010). The physiology of world-class sprint skiers. Scand. J. Med. Sci. Sports.

[6] Saltin B., Strange S. (1992). Maximal oxygen uptake: “old” and “new” arguments for a cardiovascular limitation. Med. Sci. Sports Exerc..

[7] Larsson P., Olofsson P., Jakobsson E., Burlin L., Henriksson-Larsén K. (2002). Physiological predictors of performance in cross-country skiing from treadmill tests in male and female subjects. Scand. J. Med. Sci. Sports.

[8] Myers J., Ashley E. (1997). Dangerous curves. A perspective on exercise, lactate, and the anaerobic threshold. Chest.

[9] Andersson E.P., Björklund G., Holmberg H., Ørtenblad N. (2016). Energy system contributions and determinants of performance in sprint cross-country skiing. Scand. J. Med. Sci. Sports.

[10] Helgerud J., Wisløff U. (1998). Methods for evaluating peak oxygen uptake and anaerobic threshold in upper body of cross country skiers. Med. Sci. Sports Exerc..

[11] Åstrand P.-O., Rodahl K. (2003). Textbook of Work Physiology: Physiological Bases of Exercise.

[12] Svedahl K., MacIntosh B.R. (2003). Anaerobic Threshold: The Concept and Methods of Measurement. Can. J. Appl. Physiol..

[13] Helgerud J., Høydal K., Wang E., Karlsen T., Berg P., Bjerkaas M., Simonsen T., Helgesen C., Hjorth N., Bach R. (2007). Aerobic high-intensity intervals improve VO2max more than moderate training. Med. Sci. Sports Exerc..

[14] Jacobs I., Svedenhag J. (1982). Changes in onset of blood lactate accumulation (OBLA) and muscle enzymes after training at OBLA. Graefe’s Arch. Clin. Exp. Ophthalmol..

[15] Mamen A., Tillaar R.V.D., Laparidis C. (2011). Precision in Estimating Maximal Lactate Steady State Performance in Running Using a Fixed Blood Lactate Concentration or a Delta Value from an Incremental Lactate Profile Test. IJASS Int. J. Appl. Sports Sci..

[16] Borg G.A. (1982). Psychophysical bases of perceived exertion. Med. Sci. Sports Exerc..

[17] Tanaka H., Monahan K.D., Seals D.R. (2001). Age-predicted maximal heart rate revisited. J. Am. Coll. Cardiol..

[18] Berglund I.J., Sørås S.E., Relling B.E., Lundgren K.M., Kiel I.A., Moholdt T. (2019). The relationship between maximum heart rate in a cardiorespiratory fitness test and in a maximum heart rate test. J. Sci. Med. Sport.

[19] Rusko H. (2003). Handbook of Sports Medicine and Science, Cross Country Skiing.

[20] Mann T.N., Lamberts R.P., Lambert M. (2013). Methods of Prescribing Relative Exercise Intensity: Physiological and Practical Considerations. Sports Med..

